# The personal and workplace characteristics of uninsured expatriate males in Saudi Arabia

**DOI:** 10.1186/s12913-017-1985-x

**Published:** 2017-01-19

**Authors:** Abdulwahab Alkhamis, Peter Cosgrove, Gamal Mohamed, Amir Hassan

**Affiliations:** 1 Saudi Electronic University, Abu Baker Al Sedge Rd, Riyadh, Saudi Arabia; 2 Liverpool School of Tropical Medicine, Pembroke Place, L3 5QA Liverpool, UK

**Keywords:** Health insurance, Saudi health insurance, Minorities and access to health insurance, Expatriates health insurance, Uninsured characteristics

## Abstract

**Background:**

A major concern by the health decision makers in Gulf Cooperative Council (GCC) countries is the burden of financing healthcare. While other GCC countries have been examining different options, Saudi Arabia has endeavoured to reform its private healthcare system and control expatriate access to government resources through the provision of Compulsory Employment-Based Health Insurance (CEBHI). The objective of this research was to investigate, in a natural setting, the characteristics of uninsured expatriates based on their personal and workplace characteristics.

**Methods:**

Using a cross-sectional survey, data were collected from a sample of 4,575 male expatriate employees using a multi-stage stratified cluster sampling technique. Descriptive statistics were used to summarize all variables, and the dependent variable was tabulated by access to health insurance and tested using Chi-square. Logistic analysis was performed, guided by the conceptual model.

**Results:**

Of survey respondents, 30% were either uninsured or not yet enrolled in a health insurance scheme, 79.4% of these uninsured expatriates did not have valid reasons for being uninsured, with Iqama renewal accounting for 20.6% of the uninsured. The study found both personal and workplace characteristics were important factors influencing health insurance status. Compared with single expatriates, married expatriates (accompanied by their families) are 30% less likely to be uninsured. Moreover, workers occupying technical jobs requiring high school level of education or above were two-thirds more likely to be insured compared to unskilled workers. With regard to firm size, respondents employed in large companies (more than 50 employees) are more likely to be insured compared to those employed in small companies (less than ten employees). In relation to business type, the study found that compared to workers from the agricultural sector, industrial/manufacturing, construction and trading sectors, workers were, respectively, 76%, 85%, and 60% less likely to be uninsured.

**Conclusion:**

Although the CEBHI is mandatory, this study found that the characteristics of uninsured expatriates, in respect of their personal and workplace characteristics have similarities with the uninsured from other private employment-sponsored health insurance schemes. Other factors influencing access to health insurance, besides employee and workplace characteristics, include the development and extent of the country’s insurance industry.

## Background

A particular concern posed by the scale of expatriate populations in Gulf Cooperative Council (GCC) countries is the burden of financing healthcare. Saudi Arabia, as with other GCC countries, has a dominant expatriate worker population as 90% of private sector workers are expatriates [1]. As a percentage of the total labour force, the expatriate^1^ percentage share in the Kingdom of Bahrain is 61.9%, Kuwait 84.8%, Oman 64.3%, Qatar 81.6%, UAE 89.8%, and Saudi Arabia 55.8% [2]. The healthcare financing systems in GCC countries are still being developed. At present, financing for most of their public services, including healthcare services, is through revenue from natural resources (i.e. oil or gas) [3]. Saudi Arabia, however, has attempted to reform its private healthcare system and reduce expatriate access to government resources through the provision of the Compulsory Employment-Based Health Insurance (CEBHI). At the same time, other GCC countries have been looking at various options for financing their healthcare services [3], but have yet to identify or implement an approach enabling them to reduce the burden of healthcare expenditure imposed by their expatriate worker populations and are at the stage of trying to learn lessons from one another’s experiences [4–8].

The Organization for Economic Co-operation and Development (OECD) distinguishes public from private insurance by the source of funds [9]. Private health insurance is often characterized as voluntary for-profit commercial coverage in contrast to mandatory, publicly financed and publicly managed insurance. Ultimately, all money comes from household or employer income, but in public insurance programs, this money is channelled through the state via general or social insurance tax, whereas the money is paid directly to the risk pooling entity in private insurance [10, 11]. The CEBHI shares the nature of its health plan with public insurance (mandatory) and shares the source of funds with private health insurance.

The phasing of the CEBHI was carried out according to the size of the firm, as with the implementation of the Korean social health insurance [12]. Health insurance in the form of the CEBHI was introduced in Saudi Arabia in 1999, but the actual implementation began on July 15, 2006, for large companies with more than 6000 employees; in September of the same year companies with more than 500 employees were included. However, the full implementation to all companies regardless of their size started on November 9, 2008. After this date, there are some expatriates who were not insured because the implementation occurred at the time for their resident permit renewal, which was not due for renewal at the time of the study. Moreover, the family members of expatriate employees who worked in a firm with more than 900 employees had to be covered by 10 May 2009. The timing of the study was critical because it fell after the implementation of the CEBHI for all expatriates, regardless of their employer’s size but did not include all the expatriates' family members.

Before the implementation of the CEBHI, the scope of medical coverage varied from one employer to another, while some employers provided full coverage - by either cash through insurers or via full reimbursement - others did not pay anything. Hence, there was no mechanism or clear method as to how the CEBHI regulations should be implemented; so although some expatriates could afford to pay for medical services, others on low incomes were rendered vulnerable to the cost of illness, due to the lack of basic healthcare and difficulty in affording out of pocket payments [13]. However, following the initial implementation of the CEBHI, it was made mandatory for all employers to participate in the scheme. The Council of The Cooperative Health Insurance (CCHI) determines the unified benefits package. The CCHI is the government body responsible for regulating and monitoring the universality of health insurance coverage [14]. Thus, all necessary examinations, treatment, medication, diagnoses and preventive procedures have been unified in the one insurance policy (see Table 1). For example, expatriate employees’ maximum co-payments are pre-determined so as not to exceed 20% of the invoice or a maximum of SR100 (USD26.67) [14]. Also, no co-insurance/deduction for inpatients service is permitted. Moreover, the unified plan covers up to $ 533.3 for dental treatments.^2^

**Table 1:** Cooperative health insurance schedule^a^ [67]

Policy coverage	Maximum benefit limit/person	Covered treatments/procedures
Maximum Benefit Limit/Person	SR 250,000	
Outpatient Treatment Expenses - Co-insurance/Deduction	0-20% per visit, Max. of SR 100 per visit	Consultations, lab tests, x-rays, medicines, medicines and other medical necessities, follow-up visits and referrals for the same illness
Physician's Fees:		
General Practitioner	SR 50	
Specialist	SR 100	
Consultant	SR 150	
Rare medical specialties	SR 250	Cardiology, brain and neurological surgery, vascular surgery, and other sub-specialties per standards of Saudi Commission for Health Specialties
Hospitalisation Expenses/Fees:		
Co-insurance/Deduction	None	
Accommodation for the patient	SR 600/day	
Accommodation for the hospital sitter	SR 150/day	
Pregnancy/Delivery Cost for married beneficiaries	SR 150,000	Shared Room (includes charges for bed, nursing, medical visits, supervision, and catering services)
Premature Babies	As per terms and conditions of the policy	Shared Room
Cost of Dental Treatment	SR 2,000	
Cost of Spectacles	SR 200	
Cost of Renal Dialysis	SR 10,000	
Cost of Acute Psychological Disorders	SR 15,000	
Corpse Repatriation to Home Country	SR 10,000	

^a^The table was amended on 4/2/2014 [68]

The insurance market in Saudi Arabia was developed in 2003. Before this, because there was opposition from some Islamic scholars, the relationship between healthcare providers and insurance was ungoverned and unsupervised [3]. They contended that in Islam, commercial insurance should not be permitted; but cooperative health insurance and not-for-profit health insurance are permissible. The Saudi Arabia constitution is based on the Holy Quran and Sunnah (Prophet Mohammed’s recorded saying), and the health insurance scheme must be linked to the constitution of the country. The term "cooperative health insurance" has been used for the CEBHI so that the required legislation is passed. However, the characteristics of cooperative health insurance do not equate with the CEBHI because the current practice is for premiums to go back to the insurance company owner as oppose to beneficiaries of the services [15].

The CEBHI scheme was implemented in Saudi Arabia to benefit expatriate workers in the private sector, with the multiple aims of regulating the provision of healthcare for expatriates (while providing financial protection against their healthcare expenses), improving utilisation of the government healthcare budget, by reducing the load on government healthcare providers, and increasing the contribution of private healthcare sector expenditure [16–18]. Indeed, according to Saudi Labour Law, employers must bear the responsibility for paying all necessary medical expenses for their expatriate employees [19].

The CEBHI scheme in Saudi Arabia differs from other forms of employer-sponsored insurance (ESI). In particular, the CEBHI scheme is compulsory, with enforcement including financial fines to be paid by employers who fail to follow the policy [14]. Moreover, expatriate workers in Saudi Arabia are unable to obtain or renew their Iqama (*residency permit*) without an official document confirming health insurance coverage for the same duration of the Iqama [14]. Also, it is not permitted for a health insurance company to reject any application for cooperative health insurance [18]. In other words, health insurance with the CEBHI is an obligation under Saudi labour law and not an employment fringe benefit. This situation contrasts with a system of voluntary employment insurance, whereby employers control both the eligibility criteria (employment status and hours worked) and who is to be offered health insurance [20]. The financial burden under ESI is largely carried by workers and their dependents, with health insurance coverage, benefits, premiums and co-payments based on an agreement between the employer and the health insurance company [21]. By implication, employees could face an increase in the premium or the co-payment, or see a reduction in the healthcare benefits of the policy. However, under the CEBHI, employers must pay the entire premium for their workers [14]. The CEBHI scheme is such that if employers do not subscribe or fail to pay the premiums of their employees, then the employer would be required to pay the premiums and a limited fine, along with losing the right to employ expatriate workers [18]. In effect, the CEBHI protects employees from the prospect of increasing costs of premiums over time; this is the opposite of the situation in, for example, the private sector in the United States, where employers are shifting the cost to their employees [22].

In summary, because of its mandatory nature and the control and regulation of financial barriers by the government, in theory at least, the CEBHI promises to guarantee access to health insurance for expatriate workers. On the other hand, the insurance sector is not well developed in Saudi Arabia since it was only established in 2003. This factor might reflect some reports which stated that employers pay insurers under the table without the employees having insurance to secure the renewal of employee residency permits [23]. However, there is little evidence on the performance of various forms of private health insurance in developing countries [24], nor is there any literature that evaluates the role of private employment-based health insurance in developing countries [25]. The objective of this paper is to investigate, in a natural ‘quasi-experimental’ setting: the characteristics of uninsured expatriates bases on their personal and workplace characteristics. It is anticipated that by drawing on Saudi Arabia’s experience of implementing the CEBHI throughout the entire country, this study will assist other GCC countries in reforming their systems of healthcare financing.

## Method

Using a cross-sectional survey, data were collected from a sample of 4,575 male expatriates. Riyadh City, the capital of Saudi Arabia, was selected as the setting for the study because the Riyadh region contains more than one-third of expatriates and one-fourth of the Saudi population [26]. A multi-stage stratified cluster sampling technique was used for the employee population. The businesses/companies of participants were identified from the Ministry of Labour database and stratified based on business type, company size and number of employees. Based on their size and economic sector, companies were randomly selected from the database. During randomization facilitated through the Statistical Package for the Social Sciences (SPSS) software, companies’ names and any related information were concealed; the only means of identification was the company’s code number, known only to the Manager, Statistics Department at the Ministry of Labour.

**Fig. 1:**
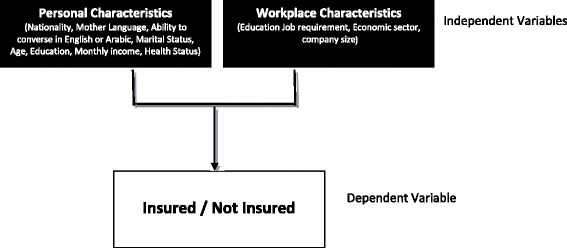
Conceptual Framework in Identifying Personal and Workplace Characteristics

Descriptive statistics were used to summarize all variables. Frequencies and percentages were calculated for the categorical variables. Mean and standard deviation values were calculated for quantitative variables. Whether people were insured or not insured was the main dependent variable - a binary variable. The dependent variable was tested using Chi-square and tabulated by access to health insurance. Logistic regression analysis was performed as guided by the conceptual model (Fig. 1). The main independent variables were based on either workplace or personal characteristics, as illustrated in Fig. 1. This analysis was used to determine the main personal and workplace characteristics of those who were not insured. The measure of association in the logistic regression was the odds ratio and its 95% confidence interval. The data collection period was from 22 May to 6 December 2010. A double data entry system was employed to minimize errors. Frequency analysis of all variables in the final data set was undertaken and all outliers were checked by revisiting the survey answers for clarification. For all of the analyses a *p*-value of less than 0.05 was considered significant.

### The Dependent Variables

The main dependent variable was whether or not expatriate male workers in the private sector had health insurance under the CEBHI scheme. This study did not measure access to health insurance based on workers having health insurance or not (as an absolute value). Accordingly, people were considered ‘insured’ if they had health insurance for a minimum of one year, the reason being that there is evidence that employers provide health insurance for a very limited period to enable acquisition of residency visa renewal [23]. Therefore, two groups were excluded from being considered insured. Firstly, those who were insured for a limited time, such as three months to get their residency visa renewal, but afterwards were not insured. Secondly, those who have been insured for less than one year were excluded from being considered insured. This group was excluded because we do not know if they will construe to be insured or not and their share of the total is small (3.3%).

The study participants were asked: ‘Have you had health insurance for at least 12 months continuously?’ For participants answering ‘No’ we provided the following options: 1) ‘My health insurance policy was valid for less than one year’; 2) ‘I am not sponsored by my company’; 3) ‘My visa is for a different job’; 4) ‘I have not renewed my Iqama’; 5) ‘I have been insured, but for less than one year’; 6) ‘I was insured for a specified length of time’, with a space provided to record the period of insurance; and 7) ‘Another reason’, with space for explanation.

### Independent Variables

As the research was focused on access to health insurance, the factors affecting expatriates’ access have been re-classified into personal characteristics and workplace characteristics (Tables 2 and 3). The socio-economic factors included in the questionnaire were based on Andersen’s Behaviour Model (1995). Socio-economic factors included were the worker’s date of birth, nationality, highest education level attained (illiterate, can read and write, completed elementary, completed high school, completed a diploma, a bachelor’s degree, a master’s degree, or doctoral studies), marital status (single or married; either married and with family living outside Saudi Arabia, or married with family living in Saudi Arabia), monthly income, comfort of the participant conversing in Arabic or English, adapted from the Medical Expenditure Panel Survey (MEPS) [27], and general questions assessing health status. Also, the questionnaire asked respondents to rate general health as being excellent, very good, good, fair or poor.

**Table 2:** Personal characteristics of respondents in the study (*n* = 4575)

		Frequency	Percent
Nationality	Non-Arab	3117	68.1
	Arab	1458	31.9
Nationalities	India	1100	24.0
	Bangladesh	1159	25.3
	Pakistan	819	17.9
	Egypt	498	10.9
	Philippines	200	4.4
	Yemen	234	5.1
	Other Arab	404	8.8
	Asian	136	3.0
	Other Nationalities	25	0.6
Can speak: (not mutually exclusive)	English	2403	52.5
	Arabic	4119	90.0
Coded Mother language	Arabic	1480	32.3
	Non-Arabic	3095	67.7
Marital status	Single/Divorced	842	18.4
	Married with accompanying family	750	16.4
	Married without accompanying family	2983	65.2
Age	<30	1006	22.0
	30-39	1895	41.4
	40-49	1289	28.2
	50-59	338	7.4
	≥60	47	1.0
	Median (Range)	36 (15.0-85.0)	
	Mean ± SD	36 ± 8.7	
Education	Illiterate	66	1.4
	Read/write	239	5.2
	Primary	1026	22.4
	Intermediate/secondary	1654	36.2
	Diploma	351	7.7
	Bachelor	1127	24.6
	Master & Doctorate	112	2.4
Monthly salary without allowance (SR)	≤2000	3085	67.4
	2001-4500	1186	25.9
	4501-6000	156	3.4
	6001-9000	97	2.1
	>9000	51	1.1

**Table 3:** The workplace characteristics of respondents in the study (*n* = 4575)

		Frequency	Percent
Type of industry	Agriculture	115	2.5
	Mining/quarrying	78	1.7
	Industrial	644	14.1
	Water and power	100	2.2
	Construction	905	19.8
	Trade	1467	32.1
	Transportation	242	5.3
	Financial/business	184	4.0
	Education and Training	692	15.1
	Other	148	3.2
Number of employees in the company	<10	725	15.8
	10-24	534	11.7
	25-50	548	12.0
	>50	2768	60.5
	Median (Range)	80 (3–40000)	
	Mean ± SD	819.5 ± 3284.7	
Education requirements of the job	Specialist with university education	1009	22.1
	Professional with education higher than high school	247	5.4
	Technical with high school education	1033	22.6
	Manual worker with less than high school education	734	16.0
	Unskilled usually with no education	1552	33.9

Workplace characteristic variables included company size, job education requirements, and economic sector. The economic sector/industry classification was based on the third revision of the International Standard Industrial Classification (ISIC) of all economic activities, which has been used to standardise the collection and reporting of statistics [28]. Each participant in the study was asked the question: ‘What is your position in your company?’ Based on the answer, the position of the participant in the company was classified according to the Ministry of Labour classifications [28]. This system was used to determine the education requirements of the job. Most expatriates in Saudi Arabia are overqualified for the jobs in which they are employed [29]. This study has adopted the Ministry of Labour classifications to enable segregation of personal characteristics (such as a worker’s education level) and workplace characteristics (the education requirements of the job).

## Results

### Descriptive Analysis

The main personal and workplace characteristics of the expatriate population are presented in Tables 2 and 3. Table 2 presents the personal characteristics of the survey respondents. The median age of expatriate workers in the private sector was 36 years. More than half (68.1%) of the expatriates working in the private sector were non-Arabs. Although two-thirds (67.7%) reported non-Arabic language as their mother tongue, the majority (90.0%) of the respondents can speak Arabic and more than half of the expatriates can speak English. Around two-thirds (67.4%) of respondents were classified as low-income earners, with less than SR2, 000 per month, excluding allowances. Additionally, just less than two-thirds (65.2%) of expatriate workers reported that they are married, although their families were not living with them in Saudi Arabia.

Table 3 presents the job and employer characteristics in which the respondents are employed. The findings show that the trading and construction sectors employed the highest proportion of expatriate workers, as more than half of the respondents were employed in these industries. One-third (33.9%) held jobs categorized for unskilled workers, usually requiring no education, whereas the respondents’ education levels revealed that less than 7.0% were illiterate or barely able to read and write. Although 60.0% of the companies randomly identified for the study employed more than 50 employees, there was a vast difference regarding employee numbers in the companies, ranging from three to 40,000 employees (with a mean of just over 800 employees and a median of 80 employees).

As can be seen in Table 4, 30% of survey respondents were either uninsured or not enrolled in a health insurance program. Moreover, 79.4% of respondents did not have valid reasons for being uninsured, while 20.6% of expatriate workers reported a valid and legal reason for not being given health insurance coverage. Specifically, that their Iqama was not due for renewal at the time of the survey being administered (Table 4). Enrolment in a health insurance program is undertaken only upon renewal of the Iqama. Also, a small percentage of the sample (3.3%) was excluded due to their being insured for less than one year.

**Table 4:** Health insurance coverage and reasons for not being insured

Health insurance status:	Frequency	Percent
0-None	1371	30.0
1-Yes for more than 12 months^a^	3053	66.7
2-Yes for less than 12 months^b^	151	3.3
Reason for not having health insurance (*N =* 1371):		
1-Had insurance but ended^c^	219	16.0
2-Sponisered by different employer	291	21.2
3-Visa was for another job^d^	148	10.8
4- I have not renewed my Iqama^e^	283	20.6
5-Insurance was done only to renew Iqama	375	27.4
6-Others (e.g. unaware of the CEBHI scheme)	55	4.0

^a^Insured continuously for more than one year

^b^Insured but did not complete a year (some people were insured for only one or two months to secure iqama renewal)

^c^The insurance of this group was terminated (some of this group were only insured for one or two months to pass the iqama newly). But when the study was conducted, they were not insured

^d^Domestic jobs (e.g. housemaid) are excluded from the CEBHI scheme and so insurance is not needed for Iqama renewal

^e^Because the study occurred less than two-years from full implementation of the CEBHI, for some expatriate workers the Iqama was not due for renewal and so they were not insured

### The Main Personal and Workplace Characteristics of Uninsured Expatriates Using Logistic Analysis

#### Marital Status

Marital status was grouped into three classes: single (reference group), married with family living with them, and married but their families are not living with them, as can be found in Table 5. Compared with single expatriates, married expatriates (accompanied by their families) are almost 30% less likely to be uninsured, (OR = 0.71, 95% CI 0.96-0.53). No significant statistical difference was found between single workers and married workers whose families are not with them in the Kingdom.

**Table 5:** Logistical Regression of uninsured expatriates based on their personal and workplace characteristics

Marital status	Odds ratio	95.0% C.I. for odds ratio		*P*-value
		Upper	Lower	
Single (Reference)				
Married without family in the Kingdom	1.03	1.4	0.77	0.565
Married with family in the Kingdom	0.71	0.96	0.53	0.026
Job educational requirement				
Unskilled worker with no education (Reference)				
Manual worker with less than high school education	0.746	1.088	0.512	0.128
Technical with high school education	0.349	0.544	0.223	0.0001
Specialist with university education	0.292	0.459	0.186	0.0001
Number of employees in the company				
<10 (Reference)				
<25	0.81	1.7	0.39	0.583
<50	0.78	1.6	0.38	0.504
≥50	0.36	0.43	0.31	0.0001
Economic sector/business type				
Agriculture (Reference)				
Industrial/manufacturing	0.24	0.39	0.15	0.0001
Construction	0.15	0.25	0.098	0.0001
Trading	0.42	0.67	0.27	0.0001
Others	0.81	1.67	0.397	0.579
Income category				
<2000 (reference)				
2000 – 4500	0.85	1.1	0.66	0.066
4501 – 6000	0.44	0.53	0.37	0.018
6001 – 9000	0.512	0.812	0.323	0.004
>9000	0.245	0.328	0.183	0.0001

#### Education Job Requirement

As outlined in Table 5, the higher the job education requirements and the lower the occurrence of being insured. Workers who had technical jobs requiring high school level education were two-thirds less likely to be uninsured compared to unskilled workers in employment with no education required (OR = 0.349, 95% CI 0.223-0.544). Compared with unskilled workers with no education, jobs requiring specialists with university education and professionals with higher than high school level education had approximately 70% less risk of being uninsured (OR = 0.29, 95% CI 0.18- 0.45). There were no statistically significant differences in the risk of being uninsured between unskilled jobs and jobs that required manual skills with less than high school education.

#### Number of Employees in the Company

Respondents employed in large companies (more than fifty employees) are two-thirds less likely to be uninsured, compared to those who are employed in small companies (less than ten employees). There was no statistically significant difference in insurance status between companies with less than ten employees and employers with less than twenty-five employees or fewer than fifty employees (see Table 5).

#### Economic Sector / Business Type

Compared to workers from the agriculture sector, industrial/manufacturing sector workers were 76% less likely to be insured (see Table 5). Construction sector workers were 85% less likely to be uninsured, compared to agriculture sector workers. Workers from the trading sector were 58% likely to be uninsured compared to agriculture sector workers. Workers from other sectors (combined) were statistically insignificant in having access to insurance as compared to agriculture sector workers.

#### Income Category

As can be found in Table 5, workers who earned more than SR 4,500 (but not more than SR 6,000) per month were around 10% less likely to be uninsured compared to workers who earned SR 2,000 or less per month. Compared to low-income earners (SR 2,000 or less), workers earning more than SR 6,000 (but not greater than SR 9,000) per month, reported being around one-third less likely to be uninsured. Workers who earned more than SR 9,000 per month were 75% less likely to be uninsured compared to low-income earners. No statistically significant difference was found between workers who earned SR 2,000 or less per month and those who earned greater than SR 2,000 (but not more than SR 45,000) per month.

## Discussion

There are similarities between the study sample and the expatriate population in Saudi Arabia. For example, the average age of the study population and the expatriate male working population in the private sector is not significantly different; the median age of the study population was 36 years old, similar to the Ministry of Labour data average of 34 years old [26]. The percentage of expatriates under 30 years old in the study sample was 22.2%, whereas the Ministry of Labour reports around 21.3% of expatriates in the same age bracket. The top six nationalities of expatriate workers in the study correspond to the top six nationalities of expatriate workers in the private sector of the Riyadh region, as per the Ministry of Labour’s database [26]. Due to the similarities between the sample size and characteristics of expatriates’ population, we are confident that the sample used for the study is representative of the expatriate population.

Saudi Arabia, like other GCC member countries, has a unique demographic composition in the private sector. Expatriate workers comprise around 90% of the total manpower in the private sector. Therefore, the burden of providing equitable access to health insurance for this group needs to be carefully considered. Specifically, major influences on workers’ access to health insurance are the characteristics of the potential recipients of the insurance and the characteristics of the providers of the insurance.

The Saudi government’s regulations designed to reduce the percentage of uninsured expatriates may not help to achieve its objectives. The regulations include the enforcement of employers to provide insurance to their expatriate workers and a unified health insurance package, along with strong government intervention through a Council and the imposition of penalties for those who fail to follow the regulations. However, this did not change the characteristics of expatriate employees who were uninsured or the employers’ characteristics because there are other influencing factors such as under-development of the health insurance industry in Saudi Arabia [3]. The health insurance companies were the greatest source of complaints in healthcare for the last eight years [30–36]. Also, there have been reports of insurers providing fake insurance to employers acquiring residency visa renewal [23].

Similar to the characteristics of uninsured workers, as documented elsewhere [37–42], the majority of uninsured expatriate workers in Saudi Arabia are young, single and categorized as unskilled and usually uneducated. More than two-thirds of expatriate workers are low income and destitute people (see Table 2). The uninsured population spans all age groups, but younger adults (19–25 years) represent 30% of the uninsured, this could be because they usually begin their careers in positions offering relatively low incomes. Saudi Arabia is similar to other countries, where the risk of being on a low income means that not only is the employer more likely to offer a job without health insurance but also that the premium is unlikely to be shared [43–45].

Studies from the United States, such as Monheit and Vistnes [46], established that a firm’s size was not an indicator of the health status of its employees but uninsured employees in both large and small firms are predictably unhealthier than insured employees [46]. However, their finding is contentious as the outcome could suggest health insurance was only offered to employees who were in good health [47]. Moreover, the present study suggests that the health status of workers in Saudi Arabia is not found to be a significant factor; this is because expatriate workers undergo rigid medical tests before deployment to their work site [48].

Furthermore, this study found that married expatriate workers have better access to health insurance due to the additional income earned by their partner. This finding is supported by a study that found the health insurance of married respondents was more related to total income as the partner’s income augments the family income [49]. Also, in Saudi Arabia, there are married expatriates with professional jobs that allow employees to bring their family with them (i.e. labour workers are not eligible to be accompanied by their family) [50]. Therefore, marital status could be reflecting job status and not marital status.

Other studies have concluded that the higher the job status and the greater the possibility employees will be insured [51–54]. Moreover, research by Chatterjee and Nielsen [55] found that there was no association between the education of expatriate workers and insurance coverage [55]. However, these studies have not considered the distinction between the job and job education requirements. Specifically, while education reflects the personal characteristics of the employee, the job education requirements reflect the importance of the job to the employer. On this basis, we investigated job and education requirements as one of the variables to assess the complexity of the job, its importance to the employers and its influence on an expatriate employee’s access to health insurance. Our study revealed a strong relationship between job requirements and insurance coverage regardless of expatriate workers’ actual education. By implication, job skills and job requirements are more important for Saudi employers when providing health insurance coverage to expatriate employees. This preferential treatment by Saudi employers in respect of health insurance can be attributed to the government’s policy of imposing conditions on the issuance of work visas. The majority of expatriate workers change their job status to ‘manual, labour’ jobs; while in their home countries, they would be in the market for employment requiring higher skills [56]. This disparity, between the workers actual education and job requirements and its influence on employers’ preference for providing health insurance, has not hitherto been studied.

As shown by studies from other countries, small sized companies are less likely to provide health insurance to their workers [45, 49, 57, 58]. The same applies in Saudi Arabia. Health insurance companies in Saudi Arabia provide cover based on risk-pooling, similar to voluntary health insurance, whereby insurers charge premiums in relation to risk [3]. However, during the sixth stage, in 2008, when the CEBHI mandated insurance for all companies, including those with less than fifty workers, insurance companies refused to participate unless their premiums were increased by 200% [59]. This finding was supported by other studies, which found, due to an increase in health insurance premiums, some companies had either stopped providing health insurance to their employees or had changed the system, imposing all or most of the contribution to their employees [60]. Also, one study in Saudi Arabia found the increase in premiums (due to high administrative costs) burdens and limits participation by small employers in the scheme [3].

Our study’s findings, relating to the influence of the economic sector on access to health insurance, are not consistent with those from other studies. We found that workers in the construction sector were more likely to be insured than workers from other sectors. However, findings from other studies suggest people in manufacturing jobs are more likely to be insured than other sectors [39, 61]. This variance could be linked to the competition among business sectors. For example, in the United States there is competition between manufacturing companies and, as a consequence, the employers provide benefits to attract workers. Alternatively, in Saudi Arabia, there is strong competition among construction companies; accordingly, employers provide an incentive package to attract workers, one of which is health insurance coverage. Construction companies also have difficulties acquiring foreign workers due to the work visa constraints set by the government. In contrast, construction companies are the largest employers by size in Saudi Arabia, whereas the manufacturing sector is the largest employer in the United States; therefore, these companies can give better health insurance benefits with a limited increase in the premium. Another study in the region found that expatriates working in construction are less likely to be insured [62]. However, this finding is inconsistent with other studies in the region where it was found that expatriates working in the construction sector were less likely to be insured. Nevertheless, it is worth noting that the study of Joshi and others did not use quantitative methods to mediate the influence of skill requirements for the job to perform the job or workers’ education from the economic sector. However, this study is consistent with other studies that show workers from the agriculture sector are less likely to be insured than those in other sectors [63].

There are other reported reasons for expatriate workers not being insured. The main reason reported by more than one-fourth (27.4%) of respondents was that insurance was provided only to renew expatriate workers’ Iqama. Our findings are supported by another study, which suggests some employers pay insurers ‘under the table’ to renew employees’ Iqamas when in fact the employees do not have health insurance [23]. This finding supports evidence that employers might play a major role in the provision of health insurance for minorities in Asia [45, 57]. The source of this behaviour could either be the employer as indicated or the employees who buy their visas from their sponsors (i.e. pay a monthly salary to their employers, to have freedom of movement) [56]. In both cases, there is supporting evidence that self-employed workers are more likely to be uninsured [39].

Our study found that the second most common reason for expatriate workers being uninsured is that employees are sponsored by different employers. These workers have legal work permits but have either ‘run-away’ from their sponsors (for various reasons) and are classified as unskilled with no education [29, 56], or work independently from their employers (mainly small company employers), who brought them to Saudi Arabia under an employer sponsorship. The employer’s role, in this case, was only to sign all legal papers of the expatriate workers [64] and receive a monthly or annual payment from their now ‘independent employees’ for this service. These employers are called labour brokers, and this service is another form of labour brokering, in which a certain sponsor brings in expatriate workers and rents them out to other companies while workers stay under the sponsorship of the labour broker. It would appear that the first form is more common in the Saudi labour market [64].

The third most common reason for expatriate workers in Saudi Arabia being uninsured was that their visa was for another job. These employees could either have illegal residence status in Saudi Arabia or are self-employed with a visa under a Saudi employer. The motivation behind this is that some expatriate workers give money to Saudi citizens to acquire visas, and pay a certain amount of money annually as a gratuity for this service. This act is illegal. There is evidence to suggest that the main incentive for Saudis to do this is financial [65]. At the end of 2013, the Saudi government undertook steps to rectify the labour market of these “labour corrections” [66]. One of the main objectives of these steps is to reduce the number of illegal workers. Further studies may be required to assess the impact of these steps to reduce uninsured expatriates.

There are some limitations to this study, one being that the study only comprised male expatriates working in the private sector; female expatriates and children were excluded. However, if gender had been included as one of the variables, it would have been very difficult to obtain sufficient participants due to the small number of female employees (98.30% of all expatriates in the private sector are male) [1]. Also, most females working in the private sector work in healthcare and all medical and non-profit sectors were excluded from this study.

One source of potential bias is the fear by study participants of recrimination from their employer, which could have resulted in invalid responses. However, an official letter and identification card from the research sponsor were provided to reassure employees that all responses would be for research purposes only, and the answers would be treated with the utmost confidentiality. In addition, research assistants administering the survey were selected from the same dominant nationalities and languages of the private sector workers, thereby ensuring that the survey could be understood and answered using the participants’ language. Finally, the study is cross-sectional, which may have increased bias with respect to the time ordering of events.

## Conclusions

Although the CEBHI seeks to mitigate some disadvantages of the voluntary ESI by requiring employers to cover all expatriate workers with unified benefits packages, there are similarities in the characteristics of the uninsured between CEBHI and other ESI schemes. Most of the literature demonstrates size and competition in the market drive employers to provide fringe benefit packages, which includes comprehensive health insurance coverage. In Saudi Arabia, there is an additional constraint on the government’s policy, which limits work visas issued according to the type of job. Along with the lack of local manpower sources, Saudi employers are obliged to devise strategies to acquire and retain expatriate workers, and they do this by offering attractive employment incentive packages including health insurance. However, this is true for only a select group of expatriate workers depending on the employer’s needs and criticality of the job in the company.

This study revealed that the size of the employer has a substantial influence on expatriates’ access to health insurance. The premium of small size employers will be critical to successful implementation of the CEBHI scheme. The decision maker has to find a way to control the high premiums for the small employers such as unifying them in one single pool.

Since the study was undertaken, the CEBHI scheme has been developed to include an expatriate worker’s family or dependents. It is expected to worsen in the future since children and women utilize health care services more than men. A further study is needed to determine the influence of this policy. Also, with the inclusion of expatriate worker`s family or dependents, it is anticipated that health insurance premiums and health care expenses will be higher than was the case when they were for the worker alone.

The study has reveal that one of the reasons for not beginning insured was due to the practice of obtaining insurance just to meet the requirement for securing a residency permit. This fact gives an indication to the policy maker of the need to increase the solvency requirement for insurance companies thereby increasing the viability of its insurance market and protecting consumers.

## Abbreviations

CCHI: Council of cooperative health insurance
CEBHI: Compulsory employment-based health insurance
ESI: Employer-sponsored insurance
GCC: Gulf cooperative council
ISIC: International standard industrial classification (ISIC)
KAIMRC: King Abdullah international medical research centre
MEPS: Medical expenditure panel survey
PHI: Private health insurance
SPSS: Statistical package for the social sciences
SR: Saudi Riyal

## Footnotes

1 The expatriate workers or migrant workers, as sometimes they are called, are categorized as minorities.

2 The minimum benefit coverage is available at the website of the Council of Cooperative Health Insurance (CCHI): www.cchi.gov.sa.
